# Laparoscopy without uterine manipulator vs. laparotomy in endometrial cancer: a retrospective study

**DOI:** 10.1590/1806-9282.20250434

**Published:** 2025-09-19

**Authors:** Selçuk Erkılınç, Sena Özcan, Ayşe Betül Öztürk, Serhan Can İşcan, Ufuk Atlıhan, Can Ata, Hüseyin Aytuğ Avşar, Tevfik Berk Bildacı, İlker Çakır

**Affiliations:** 1 İzmir Democracy University, School of Medicine, Buca Seyfi Demirsoy Education and Research Hospital, Department of Gynecologic Oncology – İzmir, Turkey.; 2 Ministry of Health, Sarıgöl Public Hospital, Department of Obstetrics and Gynecology – Manisa, Turkey.; 3 Ministry of Health, Muş Public Hospital, Department of Obstetrics and Gynecology – Muş, Turkey.; 4 Isparta City Hospital – Isparta, Turkey.; 5 Manisa Merkezefendi Public Hospital, Department of Obstetrics and Gynecology – Manisa, Turkey.; 6 İzmir Tınaztepe University, School of Medicine, Department of Gynecology and Obstetrics – İzmir, Turkey.

**Keywords:** Endometrial cancer, Laparoscopy, Laparotomy

## Abstract

**OBJECTIVE:**

The objective of the study was to evaluate perioperative and oncologic outcomes of laparoscopy without the use of uterine manipulators and laparotomy in high-grade and serous endometrial cancer.

**METHODS:**

Patients with grade III endometrioid adenocarcinoma and serous carcinoma between 2018 and 2022 were included in the study. Preoperative staging with positron emission tomography/computed tomography or thoracoabdominal computed tomography and pelvic magnetic resonance imaging was performed. All patients underwent staging surgery including hysterectomy, bilateral salpingo-oophorectomy, peritoneal washing, omentectomy, and pelvic and paraaortic lymphadenectomy up to the renal vein. No uterine manipulator was used for laparoscopic hysterectomy. Age, CA 125 level, body mass index, accompanying diseases, pathologic data including stage, lymphovascular invasion, number of pelvic and paraaortic lymph nodes, and surgical data including surgical time, surgical complications, and adjuvant therapies were collected from the hospital database retrospectively.

**RESULTS:**

Notably, 89 patients were included in the study: 34 underwent laparotomy and 55 underwent laparoscopy. Surgical times were similar between the groups. The mean pelvic lymph node count in the laparotomy and laparoscopy groups was 33 and 34, respectively. The mean paraaortic lymph node counts in the laparotomy and laparoscopy groups were 23 and 22, respectively. Red blood cell transfusion, hemorrhage, urinary tract infection, postoperative fever, bladder atony, bladder injury, and chylous leakage showed no significant differences between the groups. However, ileus, intestinal injury, and evisceration were significantly higher in the laparotomy group. Hospital stay was significantly longer in the laparotomy group compared with the laparoscopy group. Overall and recurrence-free survival were similar between the groups.

**CONCLUSION:**

Laparoscopic surgery, performed without manipulators, provides comparable oncologic outcomes to open surgery in the treatment of high-grade endometrial cancer, while also offering improved perioperative results.

## INTRODUCTION

Endometrial cancer is the most common malignancy in women. Surgery is the mainstay of treatment for endometrial cancer. Although sentinel lymph node emerged as a less radical surgical method for endometrial cancer surgery, the use of sentinel lymph nodes for high-grade endometrial cancer among gynecologic oncologists is not standard. The accuracy of sentinel lymph nodes has been reported to be as high as 90%^1^. However, the lack of information on the long-term outcomes for patients who undergo sentinel lymph node mapping necessitates prudent use of this method in cases of high-grade endometrial cancer. Peritoneal dissemination is frequently observed in serous tumor laparoscopy (LS), warranting cautious application of this method in high-grade cases. Current literature indicates that minimally invasive surgery for high-grade endometrial cancer is safe, demonstrating comparable recurrence-free survival and similar operative outcomes^2^. The safety of minimally invasive surgery in endometrial cancer has generally been investigated in low-risk patients, and the LACE study demonstrated that minimally invasive surgery yielded similar outcomes to open surgery in low-risk patients^3^. The GOG LAP-2 study is a larger-scale prospective randomized controlled trial conducted in this field. In contrast to the LACE study, a small portion of the patients in this study constituted high-risk patients, and similar to the LACE study, LS was found to be no different from laparotomy (LT)^4^. There are also studies in the literature comparing LT and LS in high-risk endometrial cancer. However, in these studies, aggressive types such as leiomyosarcoma and stromal sarcoma were included, making it difficult to isolate the effect of high-grade endometrial adenocarcinoma^5^. Therefore, in our study, we aimed to compare LS without the use of a uterine manipulator and LT in high-grade endometrial cancer, which remains a topic not yet fully elucidated in the literature.

## METHODS

Patients who underwent staging surgery between 2018 and December 2022 were included in the study. This retrospective study was approved by the institutional review board (2023/8-158). Patients with high-grade endometrial cancer and endometrioid adenocarcinoma grade III that achieved R-0 resection were included in the study. Patients with concomitant ovarian cancer were excluded. Patients with pelvic or peritoneal dissemination and those who underwent hysterectomy without lymphadenectomy were also excluded. Endometrial cancer was diagnosed through endometrial biopsy. Serous carcinoma and high-grade endometrial cancer were reported by experienced gynecologic pathologists using morphologic and immunohistochemistry analysis. Preoperative positron emission tomography (PET/CT) or thoracoabdominal computed tomography (CT) was performed to eliminate distant metastasis. Preoperative pelvic magnetic resonance imaging (MRI) in all patients to assess myometrial invasion and guide surgical decision-making. Surgical approach (LS vs. LT) was determined according to the attending surgeon's preference. A full staging procedure including hysterectomy, salpingo-oophorectomy, pelvic and paraaortic lymphadenectomy up to the renal vein, omentectomy, peritoneal washing, and peritoneal biopsy was performed in both groups. Laparoscopic paraaortic lymphadenectomy was performed using the extraperitoneal approach. The removal of presacral, precaval, paracaval, preaortic, interaortocaval, and left paraaortic lymph nodes was performed in both groups.

Pelvic lymphadenectomy was performed using the transperitoneal approach in the LS group. The borders of the pelvic lymphadenectomy were the deep circumflex iliac artery caudally, the obliterated umbilical artery medially, the bifurcation of the common iliac artery cranially, and the obturator nerve dorsally. An infracolic omentectomy was performed in all patients included in the study. A vertical midline incision from the xiphoid process to the pubis was made for staging surgery during LT. All patients were investigated for suspected intraabdominal or pelvic peritoneal dissemination. In the LS group, no uterine manipulators were used. In this technique, both ureters were lateralized starting from the pelvic brim. For colpotomy, a vaginal retractor was covered with a latex surgical glove to avoid thermal injury from the monopolar electrocautery device. The latex-covered retractor was inserted vaginally and used as a guide to delineate the circumferential colpotomy incision. The decision on whether adjuvant therapies such as radiotherapy and chemotherapy would be administered was made by a tumor board consisting of radiation oncologists, medical oncologists, and a gynecologic oncologist. Demographic data including age, CA 125 levels, body mass index (BMI), gravida, accompanying diseases, preoperative imaging, tumor diameter, myometrial invasion, surgical time, International Federation of Gynaecology and Obstetrics (FIGO) stage, number of pelvic and paraaortic lymph nodes, lymphovascular space invasion, cervical involvement, adjuvant chemotherapy and radiotherapy, surgical complications including bowel and bladder complications, pulmonary complications, postoperative vault complications, urinary tract infections, major and minor hemorrhage, emphysema, conversion to laparotomy, hospital stay, and recurrence were collected retrospectively.

Continuous data were compared using the independent sample t-test and the Mann-Whitney U test where suitable. Categorical data were compared using the chi-square test. Fisher's exact test was used when expected value problems occurred. Univariate and multivariate Cox regression was performed. For survival estimation and the comparison of survival between the LS and LT groups, Kaplan-Meier survival analysis was performed. Log-rank p-values were used for the detection of statistical significance. p<0.05 were regarded as statistically significant.

## RESULTS

A total of 89 patients were included in the study: 34 underwent LT and 55 underwent LS. The mean age was 62 years in the LT group and 60 in the LS group. The median CA 125 levels were 7 and 6 in the LT and LS groups, respectively. Morbid obesity (BMI>40 kg/m^2^) was significantly higher in the LS group (78.2 vs. 50%, p=0.01). Preoperative tumor diameter was 3.9 cm in the LT group and 2.9 cm in the LS group. Serous carcinoma was diagnosed in 51.4% of patients in the LT group and 60% of those in the LS group; the remainder had grade III endometrioid adenocarcinoma. Deep myometrial invasion on imaging was detected in 42.9% of patients in the LT group and 36.4% of patients in the LS group. The comparison of surgical outcome data is shown in Table 1.

**Table 1:** Demographic and pathological data for high-grade endometrial cancer and serous carcinoma in laparoscopy and laparotomy groups.

		Laparotomy	Laparoscopy	p-value
Age		62±12	60±10	0.501
Gravida		3 (0–5)	3 (0–7)	0.266
Preoperative				0.514
Histology				
	Endometriod III	17 (48.6)	22 (40)	
	Serous	18 (51.4)	33 (60)	
Operation time		4.9±1.9	3.9±1.9	0.377
Myometrial invasion		20 (57.1)	25 (45.5)	0.387
LVI		15 (42.9)	5 (9.1)	<0.001
Pelvic LN		33±19	34±18	0.771
Paraaortic LN		23±20	22±18	0.741
Cervical involvement		13 (37.1)	9 (16.4)	**0.009**
BMI>40		17 (50)	43 (78.2)	**0.01**
Intestinal injury		3 (7.5)	1 (1.8)	0.154
Evisceration		4 (11.8)	0 (0)	**0.019**
Pelvic abscess		1 (2.9)	1 (1.8)	1.000
Ileus		(14.7)	0 (0)	**0.007**
Atelactasis		4 (11.8)	0 (0)	0.124
Chylous leakage		6 (17.6)	10 (18.2)	1.000
Transfusion of RBC		0 (0–4)	0 (0–2)	0.502
Minor bleeding		9 (25.7)	10 (18.2)	0.393
Major bleeding		2 (5.7)	0 (0)	0.149
Urinary infection		7 (20)	7 (12.7)	0.384
Fever		3 (8.6)	2 (3.6)	0.294
Bladder atony		2 (5.7)	1 (1.8)	0.334
Bladder injury		1 (2.9)	1 (1.8)	1.000
Vault hematoma		3 (.8.6)	5 (5.5)	0.432
Emphysema		0 (0)	4 (7.3)	0.154
Stage				
	1A	8 (22.9)	27 (49.1)	**<0.001**
	1B	10 (28.6)	18 (32.7)	
	2	5 (14.3)	5 (9.1)	
	3 A	0 (0)	0 (0)	
	3C1	5 (14.3)	2 (3.6)	
	3C2	7 (20)	3 (5.5)	
Hospital stay		14.8±11	8.9±4.8	**0.001**

LVI: lymphovascular involvement; LN: lymphadenectomy; RBC: red blood cell; BMI: body mass index. Statistical values are given in bold.

Although surgical time was longer in the LT group, no statistical difference was found. Surgical complications, including transfusion, hemorrhage, infections, bladder dysfunction, and vault hematoma, were comparable. All LS procedures were completed without the need for conversion to LT. The mean pelvic and paraaortic lymph node counts were similar. Intestinal serosal injury was observed in 7.5% of patients in the LT group and 1.7% of those in the LS group, and intestinal evisceration occurred in 11.8% of patients in the LT group. Ileus was significantly higher in the LT group (14.7 vs. 0%, p=0.007). Hospital stay was significantly longer in the LT group. Postoperative atelectasis and chylous leakage rates were comparable. Stage IIIC disease was more frequent in the LT group.

**Figure 1:**
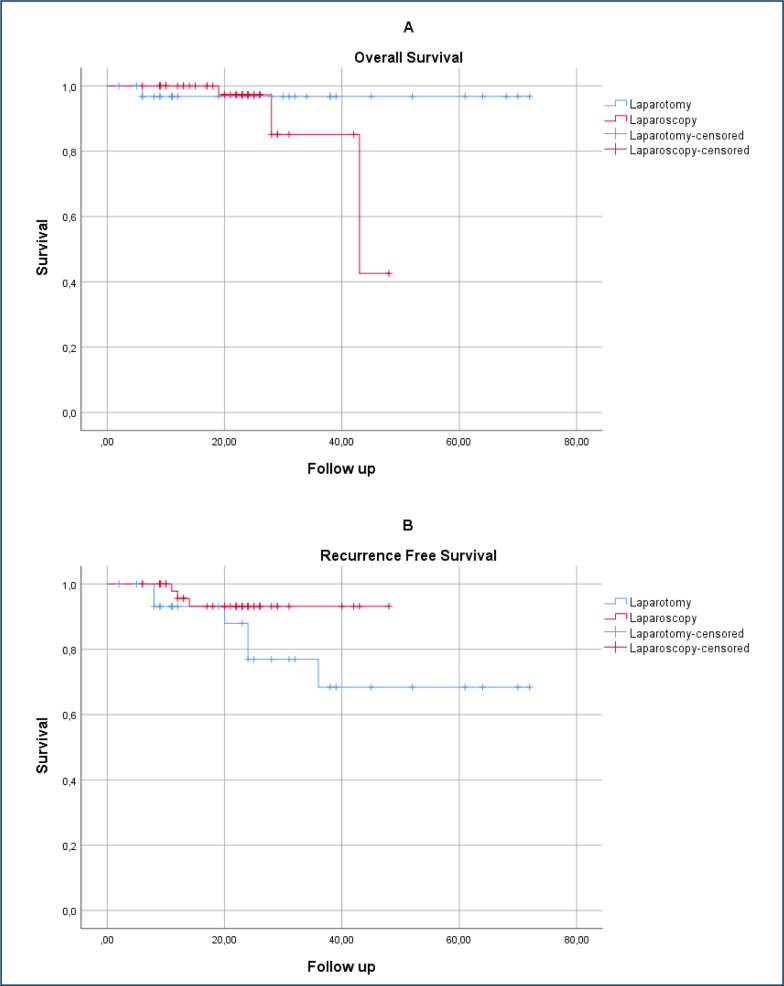
Overall survival and recurrence free survival in patients underwent laparoscopy and laparotomy.

CA 125 was the only parameter affecting survival in univariate analysis, but it was not significant in the multivariate model. Surgery type did not influence survival (HR 1.89, 95%CI 0.12–27.5; p=0.068). The Cox regression analysis is presented in Table 2. Surgical time was the only independent predictor of overall complications (OR 1.95, 95%CI 1.23–3.11). Although LS appeared protective in univariate analysis, it was not significant in multivariate analysis (Table 2). The overall and recurrence-free survival rates were similar between the groups (log-rank test showed no statistically significant difference, p>0.05), as shown in Figure 1.

**Table 2:** Cox regression model for overall survival in high-grade and serous endometrial carcinoma and risk factors for overall complications in patients with high-grade and serous endometrial carcinoma.

		Overall survival			Complications	
		HR (95%CI)	p		HR (95%CI)	p
Ca 125		1.0 (0.98–1.02)	0.647	Age	0.99 (0.95–1.03)	0.683
Age		1.0 (0.98–1.11)	0.525	HT	0.99 (0.9–1.0)	0.998
Radiotherapy			0.693	DM	0.56 (0.19–0.16)	0.284
	None	Reference				
	Yes	0.58 (0.04–8.5)				
BMI			0.437	Time for surgery	1.83 (1.25–2.68)	**0.002**
	<40	Reference				
	>40	0.89 (0.67–1.18)				
Route of surgery			0.068	BMI	0.96 (0.89–1.03)	0.292
Open		Reference				
Laparoscopy		1.89 (0.12–27.5)				
				Route of surgery	0.27 (0.11–0.70)	**0.007**

HR: hazard ratio; CI: confidence interval; BMI: body mass index; DM: diabetes mellitus; HT: hypertension. Statistical values are given in bold.

## DISCUSSION

We found that the surgical complication rates were comparable, and, moreover, the radicality of the surgery did not differ. Additionally, the survival rates were similar, and the hospitalization duration was shorter, and postoperative complications were fewer in the LS group.

High-grade endometrial cancer is a disease that typically affects patients of advanced age^6^. The patients included in the study were in advanced age. Surgical treatment of older patients can be challenging for surgeons. High-grade endometrial cancer necessitates comprehensive staging^7^. Considering the presence of additional comorbidities in these patients, the association of endometrial cancer with obesity, and the systemic nature of cancer, and radical surgeries for cancer treatment are likely to increase morbidity. It has been demonstrated that all potential complications following endometrial cancer surgery are correlated with the frailty of patients, especially in those who undergo LT^8^. In the research, one-third of the patients underwent LT, and it was found that LT increased the hazard ratio for all complications by sevenfold^7^.

Surgeons may choose the surgical method for managing endometrial cancer in non-obese patients based solely on oncologic safety. However, when considering cases like our patient group with morbid obesity, increased wound complications should be taken into account in the decision-making process. The LS approach is almost a standard method if the technology and sufficient expertise are available because the majority of endometrial cancers are detected at an early stage, and hysterectomy with sentinel lymph node sampling is now the standard treatment for most patients^9^. Many studies have shown that lymphadenectomy does not provide a survival advantage in endometrial cancer^10^. However, there are no randomized controlled trials regarding the effect of lymphadenectomy when suspicious lymph nodes are present or in the case of high-grade histology in endometrial cancer.

The SENTOR and FIRES studies evaluated the performance of sentinel lymph nodes in high-grade endometrial cancer and concluded that due to the low incidence of isolated paraaortic lymph node involvement, paraaortic lymphadenectomy might be abandoned in patients with high-grade histology if the sentinel lymph node algorithm was followed. In our study, a higher rate of paraaortic lymph node positivity was observed in both the LS and LT groups compared with these two studies. The low rates found in the SENTOR and FIRES studies should be retested in future studies. If adequately staged, serous carcinoma has been reported to have an extrauterine spread rate of around 40%. Current literature emphasizes the need for prospective studies to investigate sentinel lymph node investigation in high-grade endometrial cancer^1^. The National Comprehensive Cancer Network (NCCN) 2024 guidelines suggested that paraaortic lymphadenectomy might be performed in high-risk patients within the uterine cancer algorithm^11^. Therefore, full staging remains the most valid approach in high-grade serous carcinoma. When LS is chosen for full staging, the key point is whether the surgery can be performed to the same extent. The GOG LAP 2 study reported a median number of 18 pelvic and seven paraaortic lymph nodes in both the LS and LT groups, whereas in our study, nearly twice the number of lymph nodes were removed compared with the numbers reported in that study^12^. The range of reported lymph node counts in the literature varies widely: pelvic lymph nodes from 7 to 36 and paraaortic lymph nodes from 3 to 17, so our study has one of the highest harvested lymph node count^13–15^. Although there are studies with a similar number of lymph nodes in the LT group, the reason for our significantly higher lymph node counts in the LS group compared with the literature is that we performed paraaortic lymphadenectomy using an extraperitoneal approach in our study.

Older individuals diagnosed with endometrial carcinoma tend to have higher rates of morbidity, so it is crucial to prioritize the exploration of the safe application of minimally invasive surgical techniques for this group^16^. In our study, conditions such as ileus, evisceration, and bowel injury were more frequently observed in the LT group, providing significant evidence in favor of choosing LS for high-grade endometrial cancer. Our study showed no difference in terms of major and minor hemorrhage. It has been shown that perioperative complications increased as the surgical time increased^17^. Longer surgical time was reported for LS in patients with endometrial cancer^18^. In our study, the lack of difference in surgical duration can be attributed to performing paraaortic lymphadenectomy via the extraperitoneal route.

There is increasing evidence that uterine manipulators may raise the recurrence rate of endometrial cancer^19,20^. We believe it is illogical to insert a medical device into cancerous tissue and disrupt the tumor, so we do not use manipulators in patients with cancer. In our study, the comparable overall survival and disease-free survival outcomes between laparoscopic and open surgery may be attributed to performing extensive surgery similar to open procedures and avoiding the use of manipulators.

This study has limitations due to its retrospective design and the heterogeneity of key patient characteristics such as stage and lymphovascular invasion. Another limitation is the lack of long-term follow-up for the patients. However, the study's strength lies in the detailed perioperative characteristics of the patients. Additionally, the fact that both open and laparoscopic surgeries were performed by the same surgeons reduces the potential for bias.

## CONCLUSION

Laparoscopic surgery without the use of manipulators yields similar oncologic outcomes to open surgery in the surgical treatment of high-grade endometrial cancer, while also demonstrating better perioperative results.

## Data Availability

The datasets generated and/or analyzed during the current study are available from the corresponding author upon reasonable request.
